# Machine Learning-Based Alexithymia Assessment Using Resting-State Default Mode Network Functional Connectivity

**DOI:** 10.3390/s25175515

**Published:** 2025-09-04

**Authors:** Kei Suzuki, Midori Sugaya

**Affiliations:** Functional Control Systems, Graduate School of Engineering and Science, TOYOSU Campus, Shibaura Institute of Technology, Research Building #14A32, 3-7-5 Toyosu, Koto-ku, Tokyo 135-8548, Japan; doly@shibaura-it.ac.jp

**Keywords:** alexithymia, source localization, default mode network, electroencephalogram, machine learning

## Abstract

Alexithymia is regarded as one of the risk factors for several prevalent mental disorders, and there is a growing need for convenient and objective methods to assess alexithymia. Therefore, this study proposes a method for constructing models to assess alexithymia using machine learning and electroencephalogram (EEG) signals. The explanatory variables for the models were functional connectivity calculated from resting-state EEG data, reflecting the default mode network (DMN). The functional connectivity was computed for each frequency band in brain regions estimated by source localization. The objective variable was defined as either low or high alexithymia severity. Explainable artificial intelligence (XAI) was used to analyze which features the models relied on for their assessments. The results indicated that the classification model suggested effective assessment depending on the threshold used to define low and high alexithymia. The maximum receiver operating characteristic area under the curve (ROC-AUC) score was 0.70. Furthermore, analysis of the classification model indicated that functional connectivity in the theta and gamma frequency bands, and specifically in the Left Hippocampus, was effective for alexithymia assessment. This study demonstrates the potential applicability of EEG signals and machine learning in alexithymia assessment.

## 1. Introduction

In recent years, there has been increasing interest in the treatment and prevention of mental disorders. According to a report by the World Health Organization (WHO), approximately 1 billion people out of the global population of 7.5 billion suffer from some form of mental disorder [1]. Another report indicated that approximately 30% of individuals are affected by mental disorders at some point in their lives [2]. Mental disorders are increasingly recognized as one of the leading causes of the global burden of disease (GBD) [3], which is used to quantify the impact on human life expectancy and mortality [4], and the economic and human costs arising from ill health [2,4]. Given the significant impact of mental disorders on human life and associated healthcare costs, early detection, timely intervention, and preventive measures are of critical importance.

Notably, treatment resistance in a subset of mental disorders poses a critical problem for healthcare costs. Treatment resistance is defined as the failure to achieve a therapeutic response despite adequate treatment, and affects 20–60% of patients with mental disorders [5,6]. This also leads to a tenfold increase in healthcare burden and costs compared to general patients [6]. To alleviate the treatment burden and economic costs on patients with numerous mental disorders, there is growing interest in the treatment and prevention of mental disorders. Therefore, addressing the causes and risk factors of mental disorders is considered important for their treatment and prevention.

In addressing the causes and risk factors of mental disorders, alexithymia has received attention. Alexithymia is a personality trait [7] characterized by difficulties in identifying and describing emotions, and it is considered one of the risk factors for mental disorders [8]. Individuals experiencing difficulties in identifying emotions, a core characteristic of alexithymia, often struggle to recognize negative emotions such as stress. This impaired emotional recognition can subsequently lead to inadequate emotion regulation [9], potentially contributing to mental disorders. For instance, delayed emotional processing with negative emotions [9,10] can lead to unintentionally prolonged exposure to negative emotions. Consequently, alexithymia can lead to the development of mental disorders such as depression and anxiety disorders. Furthermore, it has been suggested that alexithymia influences the outcomes of mental disorder treatment [11]. Given these considerations, alexithymia has garnered interest due to its role as a risk factor for mental disorders and its contribution to predicting treatment outcomes.

While alexithymia has received considerable attention, its assessment has limitations in terms of objectivity and convenience. Current assessment methods involve self-report questionnaires completed by individuals and interviews conducted by clinicians [7]. These methods have demonstrated a certain degree of validity. In particular, self-report questionnaires are the most widely used assessment methods [7]. However, the validity of these questionnaires for assessing one of alexithymia’s core components has been questioned [7]. Furthermore, these self-report questionnaires tend to lead to subjective assessments; thus, objectivity is restricted. In addition, the interviews tend to consume time [12], so their convenience is restricted. Therefore, we aimed to improve the objectivity and convenience of alexithymia assessment.

To improve the objectivity and convenience of assessment, evaluation using electroencephalogram (EEG) signals with machine learning has been proposed in mental disorders related to alexithymia [13,14]. EEG signals are quantitative recordings of brain activity, reflecting the brain’s processes that give rise to human thoughts and personality. Additionally, as EEG signals are quantitatively recorded, they are more objective than qualitative human assessments. Moreover, compared to other brain activity recording methods such as positron emission tomography (PET) and functional magnetic resonance imaging (fMRI) [15], EEG signals can be measured more conveniently [16]. As these EEG signals are learned by machine learning models, the models can then objectively assess alexithymia according to their algorithms. If machine learning models could extract and leverage beneficial information from complex data that human analysis struggles to grasp, it could facilitate more convenient and more objective diagnostic support [14]. This approach, which leverages the convenience and objectivity of EEG signals and machine learning, has achieved notable success in the evaluation of mental disorders [13,14]. In addition, employing explainable artificial intelligence (XAI) techniques, which have advanced significantly in recent years, allows for the analysis of factors a model uses for its evaluations [17]. This analysis can yield new insights and deepen understanding. Thus, an approach that evaluates alexithymia using machine learning and EEG signals could offer both objectivity and convenience in its assessment.

In related work, approaches other than EEG have been explored to estimate alexithymia. Farhoumandi et al. attempted to estimate alexithymia from the scores of facial emotion recognition tasks. Their approach was based on reports of reduced activity in brain regions involved in extracting emotional information from facial expressions in individuals with alexithymia. Their results demonstrated that facial expression scores contribute to the estimation of alexithymia [12]. Filippou et al. reported a high-performance model using a multimodal approach that included heart rate, skin conductance, and facial electromyography [18,19]. Edwards et al. attempted estimation using multiple questionnaires that measured the degree of positive and negative emotions, emotion regulation ability, and self-as-context, among other factors. They suggested that the relationship between emotion regulation and alexithymia is mediated by the degree of self-as-context [20]. Wen et al. attempted to estimate the degree of alexithymia from the levels of social support and resilience, which is the ability to adapt and recover from adversity, as assessed by questionnaires. They suggested that these factors could be protective against alexithymia [21]. These studies have implications for the robust estimation of alexithymia and for providing support to individuals with alexithymia. On the other hand, estimation using EEG signals, which reflect brain activity responsible for human thought and mind, may enable a more biologically plausible assessment. Moreover, applying XAI to these EEG-based models could provide new biological insights.

Therefore, this study aims to construct a model predicting alexithymia using machine learning and EEG signals. Previously, there were some obstacles to constructing a model. For example, recruiting individuals with alexithymia and acquiring EEG data were time-consuming and laborious. However, the recent availability of public datasets has alleviated these difficulties, enhancing the feasibility of constructing a model.

When constructing a model using EEG signals, it is essential to utilize EEG signals that reflect the characteristics of alexithymia. This is because the machine learning estimation is performed based on these signals, and if they do not reflect the characteristics of alexithymia, the model’s performance will be degraded. To this end, this study focuses on functional connectivity within the resting-state default mode network (DMN). Resting-state EEG signals reflect the activity of several brain regions within the DMN, a network that becomes active when individuals are at rest [21,22]. Functional connectivity is a quantitative index that measures the degree of synchrony among multiple EEG signals [23]. This resting-state functional connectivity reflecting the DMN has been reported to correlate with alexithymia severity in relevant studies [22]. Therefore, it is considered that if a machine learning model learns this correlation, it could become effective for assessment. However, it remains incompletely understood whether this resting-state functional connectivity is effective for alexithymia assessment using machine learning.

To summarize this study, we propose and validate a method for constructing models to evaluate alexithymia using EEG signals and machine learning. When developing the dataset for model construction, functional connectivity was calculated from resting-state EEG data reflecting the DMN. To compute these features, signals from DMN-related brain regions were derived via source localization. Subsequently, the functional connectivity was calculated from these localized signals.

As our contributions and the results of this study, the classification model predicting low/high alexithymia achieved a maximum receiver operating characteristic area under the curve (ROC-AUC) of 0.70. This indicated that binary classification of low/high alexithymia is feasible using resting-state functional connectivity. Furthermore, analysis of the constructed model using XAI suggested that functional connectivity in the theta and gamma frequency bands and the Left Hippocampus is effective for alexithymia evaluation. These results revealed the effective frequency bands, brain regions, and models for alexithymia evaluation. To the best of our knowledge, these findings are unique contributions of this study.

The subsequent sections are organized as follows. Section 2 describes the methodology, including the proposed method and the dataset used for its validation. Section 3 describes the results and discussion of the model validation and the XAI analysis. Section 4 concludes this study.

## 2. Methodology

### 2.1. Publicly Available Dataset Used in This Study

For data analysis, we utilized the publicly available Leipzig Study for Mind–Body–Emotion Interactions (LEMON) dataset [24]. This dataset comprises data from a total of 227 participants [24], consisting of a younger group (*N* = 153, 25.1 ± 3.1 years old, range 20–35 years, 45 females) and an older group (*N* = 74, 67.6 ± 4.7 years old, range 59–77 years, 37 females). Data collection for this dataset adhered to the Declaration of Helsinki, and the research protocol was approved by the Ethics Committee of the Medical Faculty, University of Leipzig. Exclusion criteria were applied to the participants. The representative criteria are as follows [24].

- History of psychiatric diseases that required inpatient treatment for over 2 weeks in the last 10 years;
- History of neurological disorders;
- Use of any chemotherapeutic or psychopharmacological medication;
- Positive drug history.

This dataset contains EEG signals and alexithymia questionnaire results relevant for this study. Data from 203 participants, for whom no missing information was observed, were analyzed. For the EEG signal analysis, the preprocessed EEG signals in the LEMON dataset were used. These data were recorded for a total of 8 min during eyes-closed resting state. For alexithymia evaluation, the results of the German version [25] of the 26-item Toronto Alexithymia Scale (TAS) [26] were used.

### 2.2. EEG Signal Recording Method in the Publicly Available Dataset

This section describes the EEG signals recording method, which is important for understanding the characteristics of the LEMON dataset. For EEG signal recording, a BrainAmp MR plus amplifier was used. A 62-channel active ActiCAP electrode system, adhering to the international 10-10 system, was utilized for electrode placement. The amplifiers and electrodes were from Brain Products GmbH (Gilching, Germany). FCz was referenced, grounding was at the sternum, and skin electrode impedance was kept below 5 kΩ. The sessions for recording EEG signals consisted of a total of 16 blocks. Each block was 60 s long. Eight blocks were in an eyes-closed (EC) state, and the remaining eight blocks were in an eyes-open (EO) state. EC and EO states were measured alternately and repeatedly [24].

Note that only EEG signals from the EC state were analyzed in this study. This is because EEG signals during the EC state are less susceptible to noise contamination from eye movements and blinks, thereby allowing for a reduction in noise-induced effects.

### 2.3. Preprocessing of EEG Signals in the Publicly Available Dataset

The recorded EEG signals were downsampled from 2500 Hz to 250 Hz and band-pass filtered within 1–45 Hz using an 8th-order Butterworth filter. Channels exhibiting frequent voltage jumps/shifts or poor signal quality were rejected as outliers after visual inspection. Similarly, data segments containing extreme peak-to-peak deflections or large bursts of high-frequency activity were identified and removed via visual inspection. After EEG signals were decomposed into components using independent component analysis (ICA), components reflecting noise related to eye movements, blinks, or cardiac activity were removed [24].

### 2.4. Source Localization of EEG Signals

In this study, source localization was performed to extract brain region signals from the preprocessed EEG signals in the LEMON dataset, which were obtained using the recording method described in Section 2.2 and preprocessed as detailed in Section 2.3.

To perform source localization of EEG signals, we used exact low-resolution brain electromagnetic tomography (eLORETA) [27]. eLORETA has been reported to have lower localization error and a higher ability to suppress less significant sources compared to other methods [28]. We utilized Python (version 3.10.0) and MNE-Python (version 1.9.0) [29] as the software for implementing eLORETA. For the magnetic resonance imaging (MRI) data required for eLORETA execution, we used the fsaverage template. Referring to a related study [22], we defined Regions of Interest (ROIs) as brain regions associated with the DMN (Table 1). Then, to represent the signal of each ROI, we extracted the signal from the single vertex closest to each ROI’s coordinates, again referencing the related study [22].

### 2.5. Calculation of Functional Connectivity

After source estimation, functional connectivity was calculated from the signals of each ROI. This calculation was performed because this index has been reported to correlate with alexithymia scores [22], suggesting its potential effectiveness in assessing alexithymia. Functional connectivity quantifies the statistical interdependence of physiological time series recorded from different brain regions [23]. We employed the weighted phase lag index (wPLI) [30], which has been reported to be robust to noise, as a measure of functional connectivity. For wPLI calculation, mne-connectivity (version 0.7.0) [31] was used as the software. For the calculation of wPLI, EEG signals were epoched into 6-s windows with a 6-s slide, and wPLI was calculated from these epochs. wPLI was calculated for several frequency bands, as shown in Table 2. In this study, functional connectivity was calculated for all possible combinations of signals from two ROIs in each frequency band.

Using the methods described above, we calculated (number of frequency bands) × (number of all possible combinations of signals from two ROIs) = 6 × C212 = 396 values per participant. These indices were then used as explanatory variables.

### 2.6. Method for Assessing Alexithymia in Public Dataset

The 26-item German version of the Toronto Alexithymia Scale (TAS)—26 [25,26] was utilized to assess alexithymia in a public dataset. This questionnaire comprises items evaluated on a 5-point Likert scale, ranging from 1 (“does not apply at all”) to 5 (“applies completely”) [24]. The responses are aggregated to assess the following four components: (1) difficulty identifying feelings, (2) difficulty expressing and describing feelings, (3) externally oriented thinking, and (4) a total score encompassing these aspects. In this study, the total score from component (4) was used to construct the objective variable.

The TAS is recognized as a psychometrically valid instrument [7]. Furthermore, its reliability was evaluated using Cronbach’s alpha [24,25], and the values for each subscale were 0.79, 0.76, and 0.63 [24]. The 0.63 is close to the lower limit of the acceptable range, but it seems to be acceptable in the context of evaluating alexithymia [32].

### 2.7. Dataset Construction

For each participant, 396 explanatory variables were extracted using the methods described in Section 2.4 and Section 2.5. Similarly, one objective variable was created for each participant from the scores of a questionnaire used to assess alexithymia, as detailed in Section 2.6. Since the analysis involved 203 participants, a table dataset of 203 × (396 + 1) was constructed.

For the classification model, thresholds were set to create binary labels indicating low or high levels of alexithymia. Scores at or below the threshold were labeled as “low,” while scores above the threshold were labeled as “high.” Since the optimal threshold for accurate classification was unknown, the threshold was varied for determining “low” and “high” in 10-percentile increments, ranging from the 20th to the 80th percentile. This resulted in seven distinct thresholds, and a binary classification model was constructed for each of them.

### 2.8. Cross-Validation

In this study, to evaluate the machine learning models, we repeatedly divided the dataset into three types: training data, validation data, and test data. Training data are the data from which the model learns. Validation data are used to tune the machine learning model’s hyperparameters. Test data are used to assess a machine learning model’s performance.

To divide the data, StratifiedGroupKFold was implemented, which splits the data K times. The primary reason for adopting this method is to maintain the independence between the training and test datasets, thereby preventing overestimation of the model’s performance. “Stratified” means that the proportion of data for each class in the overall dataset is preserved as much as possible in the divided datasets. “Group” means that data belonging to the same group will not be distributed across both divided datasets. In this study, “the same group” refers to data from the same participant. This ensures data independence by preventing data from the same participant from being included in both the training and test data, which in turn prevents overestimation of the model’s performance. In this study, we set K = 5. For the implementation of StratifiedGroupKFold, Scikit-learn (version 1.6.1)’s [33] StratifiedGroupKFold was utilized.

### 2.9. Dataset Preprocessing

To robustly standardize the scale of the explanatory variables, scaling was performed using the median and interquartile range. Additionally, for learning, feature selection was conducted to identify and remove redundant explanatory variables based on their correlations. The threshold for the correlation coefficient was set at 0.95. For scaling, Scikit-learn (version 1.6.1)’s RobustScaler [33] was used. For feature selection, Feature-engine (version 1.8.3)’s SmartCorrelatedSelection [34] was used.

### 2.10. Model Construction

When constructing the classification model, we recognized the potential for imbalanced data across different classes. To address this, a combined approach of undersampling with bagging was employed to integrate multiple models. Undersampling is a technique [35] that balances class data by randomly removing samples from the majority class in the training dataset. Bagging involves the following steps: (1) creating new datasets by randomly sampling data with replacement from the original dataset; (2) training a model on each new dataset; (3) repeating steps 1 and 2 multiple times to construct multiple models [35]; (4) constructing an integrated model that estimates classes based on majority voting or weighted voting using multiple models. This combined approach, integrating undersampling and bagging [36], is widely used for imbalanced datasets where class distributions are imbalanced [37]. Hereafter, we refer to this method as UB. For the implementation of UB, BalancedBaggingClassifier from Imbalanced-learn (version 0.13.0) [38] was used.

We considered other well-known techniques for imbalanced data, such as the synthetic minority over-sampling technique (SMOTE) and its numerous variants [39]. However, we deliberately chose not to employ these methods in this study. Our research planned to use XAI to identify the actual physiological features used by the model for evaluation. Using SMOTE introduces synthetic data points that may not correspond to actual physiological measurements, which may impair the interpretability of XAI results.

For our classification models, six representative machine learning algorithms were employed: (1) logistic regression (LR); (2) a model that integrates multiple LR models using UB (hereafter referred to as LR UB); (3) a support vector machine (SVM) for classification with a linear kernel (hereafter referred to as SVM C(linear)); (4) a model that integrates multiple SVM C(linear) models using UB (hereafter referred to as SVM C(linear) UB); (5) an SVM for classification with a radial basis function (RBF) kernel (hereafter referred to as SVM C(RBF)); and (6) an RF for classification (hereafter referred to as RF C). These models were implemented using Scikit-learn (version 1.6.1) [33] and Imbalanced-learn (version 0.13.0) [38].

To briefly describe the characteristics of the primary models used in this study, the LR model incorporates both L1 regularization, as in Lasso regression, and L2 regularization, as in Ridge regression. The L1 regularization is expected to enable feature selection, while the L2 regularization is expected to improve stability and performance [40]. The SVM is widely recognized for its high generalization performance [41]. A linear kernel enables the model to learn linear patterns, whereas an RBF kernel allows it to capture non-linear relationships. The RF model is known to prevent overfitting, thereby achieving high generalization performance [42].

The hyperparameters for both the regression and classification models were tuned using Bayesian optimization, which was implemented with Optuna (version 3.5.0) [43]. During this optimization process, the hyperparameters were adjusted to achieve optimal performance on the validation data, ensuring it remained independent from the test data. The classification models were tuned to maximize the ROC-AUC. Table 3 displays the tuned hyperparameters for each model.

### 2.11. Model Performance Metrics

The ROC-AUC represents the area under the ROC curve. The ROC curve is plotted on a graph with the false positive rate (FPR) on the *x*-axis and the true positive rate (TPR) on the *y*-axis. This curve is generated by varying the classification threshold applied to the probabilities estimated by the classification model. The ROC-AUC ranges from 0 to 1. A value of 0 indicates that all classifications are incorrect, 0.5 indicates performance equivalent to random chance, and 1 indicates all classifications are correct [44].

### 2.12. Statistical Significance Evaluation of Machine Learning Model Performance

To statistically assess whether the performance of our trained machine learning models was higher than what could occur by chance, a permutation test [45,46] was conducted. In this study, we performed the permutation test as follows:

1. Train the machine learning model using the training data.
2. Evaluate the performance of the trained machine learning model on the test data.
3. Randomly permute the objective variables in both the training and test data.
4. Train the machine learning model using the permuted training data.
5. Evaluate the performance of the machine learning model using the permuted test data.
6. Repeat steps 3–5 an arbitrary number of times.
7. Obtain the empirical null distribution by aggregating the accuracies calculated repeatedly in step 6.
8. Evaluate the statistical significance of the performance calculated in step 2, based on the empirical null distribution obtained in step 7.

In this study, the number of repetitions for step 6 of the permutation test was set to 100 for regression models and 1000 for classification models.

Inspired by [46], an intuitive explanation of the permutation test is as follows: if the performance calculated in step 2 is higher than the 95th percentile of the accuracies that could occur by chance (obtained in step 7), then the original classification is considered statistically significant with *p* < 0.05.

### 2.13. Interpreting Machine Learning Models with Permutation Feature Importance

We analyzed which features contributed to the evaluation using permutation feature importance (PFI). PFI quantifies the degree of each feature’s contribution to a model’s performance. This value for each feature is calculated by assessing the impact on model performance after permuting that feature. If the model’s performance decreases after shuffling a feature’s values (a column in tabular data), that feature is considered important. Conversely, if shuffling a feature’s values does not change the model’s performance, the feature is considered unimportant. In this study, we calculated PFI using the following steps:

1. Train the machine learning model using the training data.
2. Evaluate the performance of the trained machine learning model on the test data.
3. Permute the data for one feature within the test dataset.
4. Evaluate the performance of the trained machine learning model using the permuted data.
5. Calculate the difference between the performance before permutation and the performance after permutation.
6. Repeat steps 3–5 ten times.
7. Average the differences in performance calculated in step 6.
8. Perform steps 3–6 for each feature.

We implemented PFI using Scikit-learn (version 1.6.1) [33].

Based on the calculated PFI, we analyzed which ROIs and frequency bands contributed to the evaluation. For ROIs, their importances were quantified by aggregating the PFI of the features derived from them. Specifically, we aggregated the data using the following steps:

1. Calculate PFI for each feature.
2. Exclude from the analysis any features with a PFI of 0 or less, as these are considered not to contribute to the evaluation.
3. Select the top n% of features based on their PFI values.
4. Count one point for the importance of the ROI from which the selected feature was derived.
5. Repeat steps 3–4, varying n at 10, 30, and 50.

ROIs that accumulate higher counts through steps 1–5 are considered to contribute more significantly to the evaluation. We quantified the importance of frequency bands using the same aggregation procedure as for the ROIs.

For example, if the wPLI of the alpha frequency band in the Left Frontal Lobe and Right Parietal Lobe contributes to the evaluation, the importance for “Frontal Lobe,” “Right Parietal Lobe,” and “alpha” would each be incremented by one. This analysis was conducted exclusively on models that demonstrated statistical significance using the method described in Section 2.12.

Although local interpretable model-agnostic explanations (LIME) and shapley additive explanations (SHAP) were considered as alternative XAI methods, this study employed PFI. The rationale for this selection is twofold: PFI can be computed within a practical timeframe, and it is capable of providing a global interpretation of the model. In contrast, while LIME is effective for local interpretations—offering explanations for individual predictions—it is not well-suited for global analysis. Furthermore, SHAP can be more computationally intensive than PFI, which raised concerns about its feasibility for the iterative calculations required in our research.

## 3. Results and Discussions

### 3.1. Classification Model Performance

Cross-validation was repeatedly performed, involving the optimization of hyperparameters using validation data, the training of the model with training data, and the evaluation of performance using test data. Table 4 presents the average performance calculated through this cross-validation. The classification model evaluated two values indicating low and high degrees of alexithymia. The thresholds defining these two values were set at every 10th percentile from the 20th to the 80th percentile. The average performance was calculated for each of these thresholds. The performance metric used was ROC-AUC.

As shown in Table 4, the classification model LR UB achieved the highest performance of 0.70 with a threshold of 20%. Additionally, the SVM C(linear) UB classification model achieved a performance of 0.67, also with a threshold of 20%. This threshold may suggest a boundary that can effectively distinguish between groups with low and high alexithymia based on resting-state functional connectivity.

### 3.2. Permutation Tests in the Classification Model

Permutation tests were performed for each of the multiple models constructed as part of the cross-validation. Table 5 presents the number of times a significant difference was observed through the permutation tests. Since the models were constructed five times through cross-validation, the maximum value for each cell in Table 5, excluding cells that represent a total sum, is 5.

Table 5 indicates that it is often challenging to classify with a performance higher than chance because the count is frequently zero. However, in cases such as the threshold of 20% that achieved the highest performance, or thresholds of 30% and 70%, some cells exhibit counts of two or three. This suggests that if the dependent variable is set with these thresholds, classification with higher performance than chance is possible. The high performance suggests that the functional connectivity of the DMN, used as a feature, contains information capable of discriminating the degree of alexithymia. Consequently, a classification into three categories using two thresholds derived from this high performance may also be a useful approach that reflects underlying biological characteristics. This could inform the development of biologically plausible models.

Furthermore, summing the counts for each classification model in Table 5 reveals relatively high counts for SVM C(linear) UB, and LR UB. These higher counts suggest that these models can achieve higher performance than by chance. These models incorporated UB, which is a countermeasure for imbalanced data, thereby suggesting the effectiveness of UB in handling imbalanced datasets.

### 3.3. Permutation Feature Importance in the Classification Model

In this study, we analyzed which ROIs and frequency bands are important for alexithymia assessment, based on PFI, which quantifies the degree of feature importance. Specifically, the analysis was conducted for classification at a 30% threshold and a 70% threshold, as the performance of the trained models often showed a statistically significant improvement in permutation tests at these thresholds.

#### 3.3.1. Important ROIs in Classification at a 30% Threshold

Table 6 presents the calculated importance of ROIs.

As shown in Table 6, the Left Frontal Lobe and Left Hippocampus exhibited higher counts in the top 10% of feature importance. Similarly, the Left Parietal Lobe and Right Parietal Lobe showed higher counts in the top 30% and 50%. These results suggest that these ROIs contribute more to the evaluation than other ROIs.

Regarding the Left Frontal Lobe, Left Parietal Lobe, and Right Parietal Lobe, it has been reported that these ROIs in individuals with alexithymia may reflect disruptions in functional connectivity and difficulties in cognitive processing and emotional labeling [47]. Therefore, it is expected that disruptions in functional connectivity within these ROIs could contribute to the assessment of alexithymia. These ROIs were suggested to contribute more to the assessment than other ROIs. Therefore, the results of this study were consistent with this expectation.

For the Left Hippocampus, there are few reports on its association with alexithymia, even when referring to a review paper [47]. Therefore, this study newly suggested that the Left Hippocampus could be useful for the assessment of alexithymia. Additionally, a related study [48] has reported a reduction in hippocampal volume in a subset of alexithymia subtypes. While a reduction in volume does not necessarily affect functional connectivity, it may suggest that the hippocampus has some influence on the assessment of alexithymia.

#### 3.3.2. Important Frequency Bands in Classification at a 30% Threshold

Table 7 presents the calculated importance of frequency bands.

As shown in Table 7, gamma band activity appeared more frequently in the top 10%, 30%, and 50% of feature importance. This suggests that gamma contributes more to the assessment than other frequency bands.

Regarding gamma band activity, a related study [49] reported that when negative emotions were presented, individuals with alexithymia did not show an increase in gamma-band functional connectivity. This finding has been reported to suggest an impairment in the processing of negative emotions by brain networks [47,49]. In contrast to this previous study [49], our study analyzed resting-state EEG signals without presenting emotion-inducing stimuli. Therefore, our findings newly suggested that gamma band activity in the resting-state, not only during stimulation, could be beneficial for the assessment of alexithymia.

#### 3.3.3. Important ROIs in Classification at a 70% Threshold

Table 8 presents the calculated importance of ROIs.

As shown in Table 8, the Right Posterior Cingulate Cortex exhibited higher counts in the top 10% of feature importance. The Left Frontal Lobe and Left Hippocampus showed higher counts in the top 30% and 50%. The Right Posterior Cingulate Cortex had higher counts in the top 50%. These results suggest that these ROIs contribute more to the evaluation than other ROIs.

Regarding the Right Posterior Cingulate Cortex, it has been reported that its functional connectivity in individuals with alexithymia is decreasing [22]. This decrease is expected to contribute to the evaluation. The results of this study suggest that this ROI contributes more to the evaluation than other ROIs. Therefore, the results of this study were consistent with this expectation.

Concerning the Left Frontal Lobe and Left Hippocampus, these ROIs were suggested to be effective for alexithymia assessment, consistent with the results in Section 3.3.1.

In contrast to Section 3.3.1, which used alternative thresholds, the Left Parietal Lobe and Right Parietal Lobe ROIs were found to be effective for alexithymia assessment. This difference suggests that the effective features for classifying alexithymia into low and high categories may vary depending on the threshold used to define these levels. This finding is considered a valuable insight when selecting an index for constructing highly accurate models. This is because if features are selected without understanding that effective indices can vary, there is a risk of decreased performance.

#### 3.3.4. Important Frequency Bands in Classification at a 70% Threshold

Table 9 presents the calculated importance of frequency bands.

As shown in Table 9, delta and theta band activity exhibited higher counts in the top 10%. Theta and alpha band activity showed higher counts in the top 30%. Alpha band activity appeared in higher counts in the top 50%. These results suggest that these frequency bands contribute more to alexithymia assessment than other frequency bands.

Regarding delta band activity, there are few reports on its association with alexithymia in the literature [47]. Therefore, this study newly suggests its potential utility for alexithymia assessment. Furthermore, a related study [50] reported differences in DMN delta band activity and functional connectivity among patients with mental disorders. The delta band activity differences reported in mental disorders may be relevant for alexithymia assessment, as alexithymia is considered one of the risk factors for mental disorders [8].

Concerning the theta band, related studies [47,51,52] have reported that individuals with alexithymia exhibit increased functional connectivity within this theta band when exposed to emotion-eliciting stimuli. This increase is considered to indicate that emotional expression and interpretation are disturbed [47]. In contrast to these related studies, our study analyzed resting-state EEG signals, which were not affected by emotion-eliciting stimuli. Therefore, the present study newly suggests the potential utility of resting-state EEG signals within the theta band for alexithymia assessment.

Regarding the alpha band, a decrease in functional connectivity among individuals with alexithymia has been reported [22]. Consequently, this decrease is expected to contribute to alexithymia assessment. As shown in Table 9, the alpha band has been suggested to contribute more to the alexithymia assessment than other frequency bands. Therefore, the results of this study were consistent with this expectation.

This study has several limitations. First, the observed associations may be influenced by confounding variables such as age and gender. Although methods such as matching can contribute to controlling for such factors, we opted to include all available data to maximize the dataset size for our machine learning model. Therefore, our results do not imply strict causal inferences and should be interpreted with caution. Second, there are concerns about the generalizability of our findings. To improve generalizability, analyzing data from multiple sources is preferable to using a single dataset, which can be susceptible to unknown biases. To enhance the generalizability within our study, we evaluated the model’s performance using a rigorous cross-validation procedure. Crucially, to avoid overly optimistic performance estimates, we ensured that data from the same participant were not shared between the training and test data, thereby avoiding the common pitfall of data leakage, which impairs model generalizability. Nevertheless, future research is required to validate these findings across diverse datasets from multiple sources.

## 4. Conclusions

The present study constructed regression and classification models to evaluate alexithymia using functional connectivity extracted from resting-state EEG signals. While the regression model aimed to assess alexithymia with finer granularity than the classification model, its construction proved challenging. The classification model was developed to assess low and high degrees of alexithymia. Its results indicated that it could effectively assess these degrees, defined by established low and high thresholds. The model achieved a maximum ROC-AUC score of 0.70. Analysis of the classification models newly suggested that the theta band, gamma band, and Left Hippocampus could be useful for alexithymia assessment.

## Figures and Tables

**Table 1:** ROI.

Index	MNI Coordinates			Anatomical Region
	x	y	z	
1	−30	40	25	Left Frontal Lobe
2	20	35	30	Right Frontal Lobe
3	−45	−15	−25	Left Temporal Lobe
4	55	−15	−20	Right Temporal Lobe
5	−5	−5	35	Left Posterior Cingulate Cortex
6	5	−10	30	Right Posterior Cingulate Cortex
7	−5	30	20	Left Anterior Cingulate Cortex
8	5	30	20	Right Anterior Cingulate Cortex
9	−5	−55	25	Left Hippocampus
10	5	−50	25	Right Hippocampus
11	−45	−50	40	Left Parietal Lobe
12	45	−50	35	Right Parietal Lobe

MNI = Montreal Neurological Institute. Note: The MNI coordinates and anatomical region are based on [22].

**Table 2:** Frequency bands.

Names of Frequency Bands	Frequency Bands (Hz)
Delta	1–3
Theta	4–7
Alpha	8–12
Beta	13–30
Gamma	31–45
1–30 Hz ^1^	1–30

^1^ The 1–30 Hz range represents a broad frequency band, distinct from other subdivided bands such as delta, theta, alpha, and beta.

**Table 3:** Tuned parameters.

Models	Hyperparameters ^1^
LR	C, l1_ratio
LR UB	C, l1_ratio, n_estimators
SVM C (linear)	C
SVM C (linear) UB	C, n_estimators
SVM C (RBF)	C, gamma
RF C	n_estimators, max_depth, min_samples_split, min_samples_leaf, max_features

LR = logistic regression; LR UB = logistic regression models applied under sampling and bagging; SVM C (linear) = support vector machine for classification with a linear kernel; SVM C (linear) UB = support vector machine for classification models with liner a linear kernel applied under sampling and bagging; SVM C (RBF) = support vector machine for classification with a radial basis function kernel; RF C = random forest for classification. ^1^ Hyperparameters column represents parameter names in Scikit-learn [33].

**Table 4:** Performance of the classification models.

Threshold ^1^	Classification Model ^2^					
	LR	LR UB	SVM C (Linear)	SVM C (Linear) UB	SVM C (RBF)	RF C
20%	0.64	0.70	0.63	0.67	0.60	0.59
30%	0.60	0.65	0.54	0.64	0.61	0.65
40%	0.48	0.52	0.44	0.54	0.48	0.49
50%	0.53	0.54	0.52	0.55	0.49	0.54
60%	0.61	0.54	0.58	0.57	0.42	0.50
70%	0.56	0.62	0.66	0.66	0.56	0.43
80%	0.56	0.49	0.52	0.55	0.50	0.55

LR = logistic regression; LR UB = logistic regression models applied under sampling and bagging; SVM C (linear) = support vector machine for classification with a linear kernel; SVM C (linear) UB = support vector machine for classification models with liner a linear kernel applied under sampling and bagging; SVM C (RBF) = support vector machine for classification with a radial basis function kernel; RF C = random forest for classification. ^1^ The values in this column represent the percentile thresholds used to define low and high levels of alexithymia, as detailed in Section 2.7. ^2^ The values in these columns represent the ROC-AUC scores for the corresponding model and threshold. The ROC-AUC score ranges from 0 to 1. A value of 0 indicates that all classifications are incorrect, 0.5 indicates performance equivalent to random chance, and 1 indicates all classifications are correct [44].

**Table 5:** Results of permutation test.

Threshold ^1^	Classification Model ^2^						
	LR	LR UB	SVM C (Linear)	SVM C (Linear) UB	SVM C (RBF)	RF C	Sum ^3^
20%	1	2	0	1	1	0	5
30%	1	3	1	3	1	1	10
40%	0	0	0	1	0	0	1
50%	0	0	0	0	0	1	1
60%	2	0	1	1	0	0	4
70%	0	1	3	3	1	0	8
80%	0	0	0	0	0	1	1
Sum ^3^	4	6	5	9	3	3	30

LR = logistic regression; LR UB = logistic regression models applied under sampling and bagging; SVM C (linear) = support vector machine for classification with a linear kernel; SVM C (linear) UB = support vector machine for classification models with liner a linear kernel applied under sampling and bagging; SVM C (RBF) = support vector machine for classification with a radial basis function kernel; RF C = random forest for classification. ^1^ The values in this column represent the percentile thresholds used to define low and high levels of alexithymia, as detailed in Section 2.7. ^2^ The values in these columns represent the number of times the model performance was found to be significantly higher than the chance level. Statistical significance was determined by a permutation test with a significance level of 0.05. This performance was evaluated five times using cross-validation. Therefore, the values range from 0 to 5. A higher value indicates that models with statistically significant performance were more consistently constructed. The detail of the methodology is described in Section 2.12. ^3^ The values in this column or row represent the corresponding total.

**Table 6:** ROI importance in classification at a 30% threshold.

ROI	Counts of Top *n*% of Feature (*n* = 10, 30, 50) ^1^		
	10%	30%	50%
Left Frontal Lobe	7	11	13
Right Frontal Lobe	0	7	10
Left Temporal Lobe	0	4	7
Right Temporal Lobe	3	7	8
Left Posterior Cingulate Cortex	3	6	7
Right Posterior Cingulate Cortex	5	8	8
Left Anterior Cingulate Cortex	3	9	9
Right Anterior Cingulate Cortex	1	8	9
Left Hippocampus	6	10	11
Right Hippocampus	3	6	7
Left Parietal Lobe	5	11	13
Right Parietal Lobe	2	11	14

ROI = Regions of Interest. ^1^ The values in these columns represent the importance of each ROI, where a higher value indicates a more influential ROI. These importance values were calculated through the following procedure: (1) Evaluate the contribution of each feature using permutation feature importance. (2) Select the top n% of features based on their permutation feature importance. (3) Count the number of ROI corresponding to these selected features. (4) Repeat steps 3–4, varying *n* at 10, 30, and 50. The methodology is described in detail in Section 2.13.

**Table 7:** Frequency band importance in classification at a 30% threshold.

Frequency Band	Counts of Top *n*% of Feature (*n* = 10, 30, 50) 1		
	10%	30%	50%
delta	8	16	20
theta	4	10	10
alpha	8	18	20
beta	6	16	18
gamma	8	24	32
1–30 Hz ^2^	4	14	16

^1^ The values in these columns represent the importance of each frequency band, where a higher value indicates a more influential frequency band. These importance values were calculated through the following procedure: (1) Evaluate the contribution of each feature using permutation feature importance. (2) Select the top n% of features based on their permutation feature importance. (3) Count the number of frequency bands corresponding to these selected features. (4) Repeat steps 3–4, varying *n* at 10, 30, and 50. The methodology is described in detail in Section 2.13. ^2^ The 1–30 Hz range represents a broad frequency band, distinct from other subdivided bands such as delta, theta, alpha, and beta.

**Table 8:** ROI importance in classification at a 70% threshold.

ROI	Counts of Top *n*% of Feature (*n* = 10, 30, 50) ^1^		
	10%	30%	50%
Left Frontal Lobe	4	12	14
Right Frontal Lobe	3	6	6
Left Temporal Lobe	4	7	8
Right Temporal Lobe	3	5	9
Left Posterior Cingulate Cortex	3	9	9
Right Posterior Cingulate Cortex	5	9	10
Left Anterior Cingulate Cortex	3	6	8
Right Anterior Cingulate Cortex	1	7	11
Left Hippocampus	3	12	12
Right Hippocampus	2	4	6
Left Parietal Lobe	1	2	3
Right Parietal Lobe	2	5	6

ROI = Regions of Interest. ^1^ The values in these columns represent the importance of each ROI, where a higher value indicates a more influential ROI. These importance values were calculated through the following procedure: (1) Evaluate the contribution of each feature using permutation feature importance. (2) Select the top n% of features based on their permutation feature importance. (3) Count the number of ROI corresponding to these selected features. (4) Repeat steps 3–4, varying *n* at 10, 30, and 50. The methodology is described in detail in Section 2.13.

**Table 9:** Frequency band importance in classification at a 70% threshold.

Frequency Band	Counts of Top *n*% of Feature (*n* = 10, 30, 50) ^1^		
	10%	30%	50%
delta	8	12	12
theta	8	18	22
alpha	4	18	28
beta	4	14	16
gamma	6	14	16
1–30 Hz ^2^	4	8	8

^1^ The values in these columns represent the importance of each frequency band, where a higher value indicates a more influential frequency band. These importance values were calculated through the following procedure: (1) Evaluate the contribution of each feature using permutation feature importance. (2) Select the top n% of features based on their permutation feature importance. (3) Count the number of frequency bands corresponding to these selected features. (4) Repeat steps 3–4, varying *n* at 10, 30, and 50. The methodology is described in detail in Section 2.13. ^2^ The 1–30 Hz range represents a broad frequency band, distinct from other subdivided bands such as delta, theta, alpha, and beta.

## Data Availability

In this study, the public dataset LEMON was analyzed.
